# Error Analysis in a Stereo Vision-Based Pedestrian Detection Sensor for Collision Avoidance Applications

**DOI:** 10.3390/s100403741

**Published:** 2010-04-13

**Authors:** David F. Llorca, Miguel A. Sotelo, Ignacio Parra, Manuel Ocaña, Luis M. Bergasa

**Affiliations:** Electronics Department, University of Alcalá, Polytechnic School, University Campus, Alcalá de Henares, Madrid 28871, Spain; E-Mails: sotelo@depeca.uah.es (M.A.S.); parra@depeca.uah.es (I.P.); mocana@depeca.uah.es (M.O.); bergasa@depeca.uah.es (L.M.B.)

**Keywords:** 3D sensors, automotive industry, computer vision, stereo quantization errors, pedestrian detection

## Abstract

This paper presents an analytical study of the depth estimation error of a stereo vision-based pedestrian detection sensor for automotive applications such as pedestrian collision avoidance and/or mitigation. The sensor comprises two synchronized and calibrated low-cost cameras. Pedestrians are detected by combining a 3D clustering method with Support Vector Machine-based (SVM) classification. The influence of the sensor parameters in the stereo quantization errors is analyzed in detail providing a point of reference for choosing the sensor setup according to the application requirements. The sensor is then validated in real experiments. Collision avoidance maneuvers by steering are carried out by manual driving. A real time kinematic differential global positioning system (RTK-DGPS) is used to provide ground truth data corresponding to both the pedestrian and the host vehicle locations. The performed field test provided encouraging results and proved the validity of the proposed sensor for being used in the automotive sector towards applications such as autonomous pedestrian collision avoidance.

## 1. Introduction

Pedestrian protection is a key problem in the context of the automotive industry and its applications. Sensor systems onboard the vehicles are required for predicting the vehicle host-to-pedestrian (H2P) distance as wells as the time-to-collision (TTC). Cameras are the most commonly used sensor for that purpose. The use of video sensors comes quite natural for the problem of pedestrian detection since they provide texture information which enables the use of quite discriminative pattern recognition techniques. The human visual perception system is perhaps the best example of what performance might be possible with such sensors, if only the appropriate algorithm is used.

Pedestrian detection is a difficult task from computer vision perspective. Large variations in pedestrian appearance (e.g., clothing, pose, *etc.*) and environmental conditions (e.g., lighting, moving background, *etc.*) make this problem particularly challenging. The first stage in most systems consists of identifying generic obstacles as regions of interest (ROIs) using prior scene knowledge (camera calibration, ground plane constraint, *etc.*) and a computationally efficient method. Subsequently, a more expensive pattern recognition step is applied. The lack of explicit models leads to the use of machine learning techniques, where an implicit representation is learned from features obtained from thousands of samples. Concerning the related work, over the last decade, a considerable number of vision-based pedestrian detection systems have been proposed. Several remarkable surveys have been presented [1–4], some of them recently published [5,6]. Most of the work concerning human motion summarized in [1–5] focuses on pedestrian protection applications in the intelligent vehicle domain, covering both passive and active safety techniques. An overview of the current state of the art from both a methodological and an experimental perspective is presented in [6], where a novel benchmark set has been made publicly available. We refer to these surveys for general and detailed background concerning pedestrian detection for automotive applications.

Accurate depth information is essential in the area of pedestrian protection applications (e.g., driving assessment, collision avoidance, collision mitigation, *etc.*). One of the advantages of stereo vision sensors is their ability to compute a detailed 3D representation of the scene around the vehicle, by passive sensing and at low sensor costs (compared with active sensors such as laser or radar). Depth information is obtained by solving the correspondence problem and performing triangulation. However, the depth reconstruction accuracy has its limitations due to the discrete nature of the stereo vision sensor. These limitations have to be analyzed to validate the sensor as a source of information for automotive applications. In addition, designing a stereo system involves choosing several parameters: the cameras focal length, the images size and the distance between the cameras (baseline). Unfortunately, a trade off must be reached between accurate depth estimation and other parameters such as the computational load and the size of the frontal blind zone. The theoretical characterization of range estimation errors based on system parameters for stereo vision is a well known topic [7,8] including applications such as robotics [9] and autonomous navigation [10]. However, these issues have been somewhat neglected in the context of pedestrian protection applications.

In this paper we present an analytical study of the depth estimation error of a stereo vision-based pedestrian detection sensor for automotive applications such as pedestrian collision avoidance and/or mitigation. The sensor comprises two synchronized and calibrated low-cost cameras. Pedestrians are detected by combining a 3D clustering method with Support Vector Machine-based (SVM) classification. The influence of the sensor parameters in the stereo quantization errors is analyzed in detail providing a point of reference for choosing the sensor setup according to the application requirements. The sensor is then validated in real experiments. Collision avoidance maneuvers by steering are carried out by manual driving. A real time kinematic differential global positioning system (RTK-DGPS) is used to provide ground truth data corresponding to both the pedestrian and the host vehicle locations. The performed field test provided encouraging results and proved the validity of the proposed sensor concerning the accuracy required in one of the most challenging and difficult applications in the context of the automotive industry.

The remainder of this paper is organized as follows: Section 2 provides an overall description of the proposed sensor, covering details of implementation, focusing on the analysis of the depth estimation error and the sensor setup and describing the proposed maneuver for pedestrian collision avoidance. Experimental results that validate the proposed approach are presented and discussed in Section 3. Finally, Section 4 summarizes the conclusions.

## 2. Stereo Sensor for Pedestrian Collision Avoidance

### 2.1. System architecture

**Figure 1.**
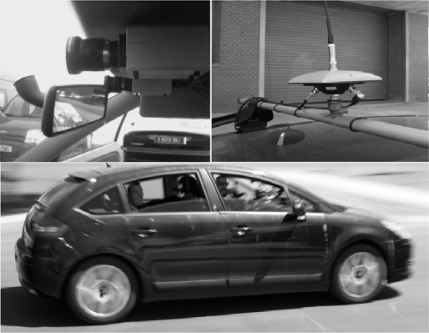
(Top left) Low cost stereo vision sensor. (Top right) RTK-DGPS. (Bottom) Experimental vehicle (modified Citröen C4).

The experimental vehicle used in this work is a car-like robot (a modified Citröen C4) which can be seen in Figure 1. It has an onboard computer housing the image processing system, a RTK-DGPS which is connected via RS232 serial port and a pair of synchronized low cost digital cameras connected via FireWire port.

The stereo vision sensor uses 320 × 240 pixel greyscale images with a baseline of approximately 300 mm and a focal length of 4.2 mm. These parameters satisfy the application requirements as we will see in subsequent sections. The cameras are calibrated in a semi-supervised fashion by using a modified version of the Camera Calibration Toolbox for Matlab and a chessboard pattern. Thus we obtain the intrinsic parameters of each camera (focal length - *f_x_*, *f_y_* -, optical centre -*u*_0_, *v*_0_-and distortion parameters -*k*_0_, *k*_1_, *k*_2_, *k*_3_ -) as well as the extrinsic transformation between them (rotation angles -*α_x_*, *α_y_*, *α_z_* - and translation vector -*t_x_*, *t_y_*, *t_z_* -). We refer to [11] for a detailed description of these parameters. Distortion parameters (*k*_0_, *k*_1_, *k*_2_, *k*_3_) are used to compensate both radial and tangential lens distortions.

### 2.2. Stereo vision-based pedestrian detection

**Figure 2.**
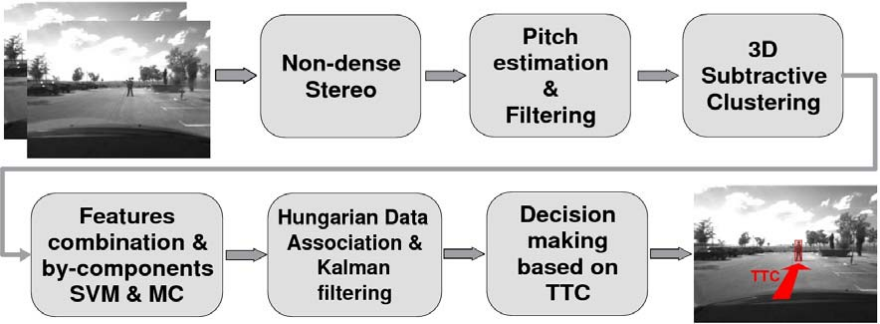
Overview of the stereo vision-based pedestrian detection architecture.

Pedestrian detection is carried out using the system described in [12,13] (Figure 2 depicts an overview of the pedestrian detection architecture). Non-dense 3D maps are computed using a robust correlation process that reduces the number of matching errors [14]. The camera pitch angle is dynamically estimated using the so-called virtual disparity map which provides a better performance compared with other representations such as v-disparity map or YOZ map [13]. Two main advantages are achieved by means of pitch compensation. First, the accuracy of the time-to-collision estimation in car-to-pedestrian accidents is increased. Second, the separation between road points and obstacle points is improved, resulting in lower false-positive and false-negative detection rates [13].

3D maps are filtered assuming the road surface as planar (which can be acceptable in most cases), *i.e.*, points under the actual road profile and over the actual road profile plus the maximum pedestrian height are removed since they do not correspond to obstacles (possible pedestrians). The resulting filtered 3D maps are used to obtain the regions of interest (ROIs).

Based on the idea that obstacles (including pedestrians) have a higher density of 3D points than the road surface, ROI selection can be carried out by determining those positions in the 3D space where there is a high concentration of 3D points. A 3D subtractive clustering method is proposed to cope with the ROI selection stage using sparse data. The idea is to find high-density regions, which are roughly modelled by a single 3D Gaussian distribution, in the Euclidean space. The parameters of each Gaussian distribution are defined according to a minimum and maximum extent of pedestrians. Thus, whereas pedestrians are correctly selected, bigger obstacles such as vehicles or groups of pedestrians are usually split in two or more parts. To cope with the stereo accuracy the method is adapted to the expected depth error [14].

The 2D candidates are then obtained by projecting the 3D points of each resulting cluster and computing their bounding boxes. A Support Vector Machine-based (SVM) classifier is then applied using an optimal combination of feature-extraction methods and a by-components approach [12]. The RBF kernel provides better performance although the linear kernel is the best solution from a computational point of view. Each candidate (possible pedestrian) is divided in six regions (head, left and right arms, left and right legs and a region between the legs). Each region is independently learnt using different features. The optimal combination is obtained using texture features (*Texture Unit Number*) for the head and the region between the legs, histograms of grey level differences for arms, and Histograms of Oriented Gradients (HOG) for the legs [12]. The final classifier is trained with 67,650 samples (22,550 pedestrians and 45,100 non pedestrians, including mirrored images).

Nonetheless, the 2D bounding box corresponding to a 3D candidate might not perfectly match the actual pedestrian appearance in the image plane. Multiple candidates are generated around each original candidate. The so-called multi-candidate (MC) approach proves to increase the detection rate, the accuracy of depth measurements, as well as the detection range [12]. The resulting pedestrians are tracked by means of a Kalman filter and the data association problem is solved using the Hungarian method.

The last block of Figure 2 is based on the computation of the time-to-collision (TTC) between the host vehicle and the pedestrians ahead and is defined according to the application requirements. For example, in case of *driving alert* applications this stage will trigger alarms to the driver depending on the host-to-pedestrians (H2P) TTC. In case of *pedestrian collision mitigation* applications [13] an activation signal will be sent to a pedestrian protection airbag and/or an active hood system. For *pedestrian collision avoidance applications* this stage will trigger the corresponding signals to the brake pedal and/or the steering wheel controllers.

The pedestrian detection system runs in real time (25 Hz) with 320 × 240 images and a baseline of *t_x_* = 300 mm approximately. The stereo vision-based pedestrian detection system has been tested in real collision-mitigation experiments by active hood triggering and collision-avoidance tests by brakeing or decelerating [13]. In this paper, its performance is analyzed in real experiments in the context of pedestrian collision avoidance by steering, including analysis of the depth estimation errors. Note that although depth estimation uncertainties have an effect in almost all blocks of Figure 2 (excluding block number 4 -*feature selection and classification*-), we will focus on the last stage by analyzing host-to-pedestrian distance estimation errors.

### 2.3. Collision avoidance maneuver by steering

Pedestrian collision avoidance is one of the most difficult and challenging automatic driving operations for autonomous vehicles and can be carried out by braking or by steering. Before designing autonomous collision avoidance maneuvers a proper analysis of the sensor errors has to be performed in order to validate the proposed approach. In our case, the stereo vision-based pedestrian detection sensor is evaluated in real scenarios with real drivers and real pedestrians. Since emergency brake maneuvers are risky, a set of experiments in which drivers have been requested to perform pedestrian collision avoidance maneuvers by steering at speeds—10, 15, 20, 25 and 30 km/h—have been devised. Higher speeds have not been considered due to the associated risks.

**Figure 3.**
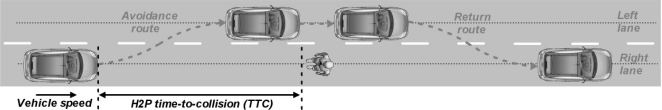
Pedestrian collision avoidance maneuver.

The avoidance maneuver has to fulfil some conditions. First, the vehicle has to be moving along a straight road in the right lane. Second, the pedestrian has to be located in the same lane. Third, the left lane has to be free and long enough for the pedestrian collision avoidance maneuver to be completed at the current speed. As soon as the driver detects a potential pedestrian collision that can be avoided, a lane change to the adjacent left lane is performed. Once the pedestrian has been passed, a second lane change is carried out to go back to the right lane (see Figure 3).

### 2.4. Stereo quantization error

There is a significant amount of published research on characterization of range estimation errors based on system parameters for stereo vision [7,8]. Here, our approach for computing the quantization error covariance for each point and the corresponding host-to-pedestrian distance estimation error is briefly described.

Given a calibrated rig of cameras and a correspondence between two points, one on the left camera (*u_l_*, *v_l_*) and another one on the right (*u_r_*, *v_r_*) the 3D position of the point in the world coordinate system is given by [11]:

(1) $$P={\left(A^{T}\;\cdot \;A\right)}^{-1}\;\cdot \;A^{T}\;\cdot \;b={\left(X,Y,Z\right)}^{T}$$

where *A* is the matrix containing the equations for the 3D to 2D transformation for each one of the cameras and *b* the independent term of the same equations. Matrices *A* and *b* are written as a function of the cameras intrinsic parameters:

(2) $$A=\left(\begin{array}{ccc} u_{l}\;\cdot \;m_{31}^{L}-m_{11}^{L} & u_{l}\;\cdot \;m_{32}^{L}-m_{12}^{L} & u_{l}\;\cdot \;m_{33}^{L}-m_{13}^{L}\\ v_{l}\;\cdot \;m_{31}^{L}-m_{21}^{L} & v_{l}\;\cdot \;m_{32}^{L}-m_{22}^{L} & v_{l}\;\cdot \;m_{33}^{L}-m_{23}^{L}\\ u_{r}\;\cdot \;m_{31}^{R}-m_{11}^{R} & u_{r}\;\cdot \;m_{32}^{R}-m_{12}^{R} & u_{r}\;\cdot \;m_{33}^{R}-m_{13}^{R}\\ v_{r}\;\cdot \;m_{31}^{R}-m_{21}^{R} & v_{r}\;\cdot \;m_{32}^{R}-m_{22}^{R} & v_{r}\;\cdot \;m_{33}^{R}-m_{23}^{R} \end{array}\right)$$

(3) $$b=\left(\begin{array}{c} m_{14}^{L}-u_{l}\;\cdot \;m_{34}^{L}\\ m_{24}^{L}-v_{l}\;\cdot \;m_{34}^{L}\\ m_{14}^{R}-u_{r}\;\cdot \;m_{34}^{R}\\ m_{24}^{R}-v_{r}\;\cdot \;m_{34}^{R} \end{array}\right)$$

Each camera intrinsic parameters [*M^L^*  *M^R^*] are estimated using an off-line calibration process. The intrinsic parameters describe the 3D to 2D transformation for each one of the cameras according to the following equation:

(4) $$\left(\begin{array}{c} \mathrm{su}\\ \mathrm{sv}\\ s \end{array}\right)=\left(\begin{array}{cccc} f/\mathrm{dx} & 0 & u_{0} & 0\\ 0 & f/\mathrm{dy} & v_{0} & 0\\ 0 & 0 & 1 & 0 \end{array}\right)\;\cdot \;\left(\begin{array}{c} X\\ Y\\ Z\\ 1 \end{array}\right)=\left(\begin{array}{cccc} m_{11} & m_{12} & m_{13} & m_{14}\\ m_{21} & m_{22} & m_{23} & m_{24}\\ m_{31} & m_{32} & m_{33} & m_{34} \end{array}\right)\;\cdot \;\left(\begin{array}{c} X\\ Y\\ Z\\ 1 \end{array}\right)=M\;\cdot \;P$$

In order to compute how the different errors in quantization in *T* = (*u_l_*   *v_l_*   *u_r_*   *v_r_*) affect the 3D position the partial derivatives for Equation (1) are computed:

(5) $$\frac{\partial (A\;\cdot \;P)}{\partial T}=\frac{\partial b}{\partial T}$$

Applying the product rule for matrices:

(6) $$P^{T}\frac{\partial A^{T}}{\partial T}+A\frac{\partial P}{\partial T}=\frac{\partial b}{\partial T}\to A\frac{\partial P}{\partial T}=\frac{\partial P}{\partial T}-P^{T}\frac{\partial A^{T}}{\partial T}$$

and writing C as:

(7) $$C=\frac{\partial b}{\partial T}-P^{T}\frac{\partial A^{T}}{\partial T}$$

the expression for how the inaccuracies in the pixel position affect the 3D reconstruction is obtained:

(8) $$A\;\cdot \;\frac{\partial P}{\partial T}=C\to \frac{\partial P}{\partial T}={\left(A^{T}\;\cdot \;A\right)}^{-1}\;\cdot \;A^{T}\;\cdot \;C$$

Solving the partial derivatives for Equation (7) using Equations (2) and (3):

(9) $$C=\frac{\partial b}{\partial T}-P^{T}\frac{\partial A^{T}}{\partial T}=I_{4\times 4}\;\cdot \;\left(\begin{array}{c} -m_{34}^{L}-m_{31}^{L}\;\cdot \;X-m_{32}^{L}\;\cdot \;Y-m_{33}^{L}\;\cdot \;Z\\ -m_{34}^{L}-m_{31}^{L}\;\cdot \;X-m_{32}^{L}\;\cdot \;Y-m_{33}^{L}\;\cdot \;Z\\ -m_{34}^{R}-m_{31}^{R}\;\cdot \;X-m_{32}^{R}\;\cdot \;Y-m_{33}^{R}\;\cdot \;Z\\ -m_{34}^{R}-m_{31}^{R}\;\cdot \;X-m_{32}^{R}\;\cdot \;Y-m_{33}^{R}\;\cdot \;Z \end{array}\right)$$

Finally, substituting the intrinsic matrices values from Equation (4):

(10) $$C=\left(\begin{array}{cccc} -Z & 0 & 0 & 0\\ 0 & -Z & 0 & 0\\ 0 & 0 & -Z & 0\\ 0 & 0 & 0 & -Z \end{array}\right)$$

Assuming T is a normally distributed random variable with mean 0 and variance:

(11) $$\sigma_{T}^{2}=\left(\begin{array}{cccc} \sigma_{u_{l}}^{2} & 0 & 0 & 0\\ 0 & \sigma_{v_{l}}^{2} & 0 & 0\\ 0 & 0 & \sigma_{u_{r}}^{2} & 0\\ 0 & 0 & 0 & \sigma_{v_{r}}^{2} \end{array}\right)$$

where $\sigma_{u_{l}}^{2}$, $\sigma_{v_{l}}^{2}$, $\sigma_{u_{r}}^{2}$, $\sigma_{v_{r}}^{2}$ are the uncertainties in pixels on the measure of *T*, the final expression for the quantization error covariance is (note that the errors in the image coordinates are assumed to be independent so the covariance matrix is diagonal):

(12) $$\begin{array}{c} \text{cov}\left(\frac{\partial P}{\partial T}\;\cdot \;T\right)=E\left[\left(\frac{\partial P}{\partial T}\;\cdot \;T\right)\;\cdot \;{\left(\frac{\partial P}{\partial T}\;\cdot \;T\right)}^{T}\right]=\frac{\partial P}{\partial T}\;\cdot \;E[T^{2}]\;\cdot \;{\left(\frac{\partial P}{\partial T}\right)}^{T}\to \\ \text{cov}\left(\frac{\partial P}{\partial T}\;\cdot \;T\right)=\frac{\partial P}{\partial T}\;\cdot \;\sigma_{T}^{2}\;\cdot \;{\left(\frac{\partial P}{\partial T}\right)}^{T}=\left(\begin{array}{ccc} \Delta_{\mathrm{xx}} & 0 & 0\\ 0 & \Delta_{\mathrm{yy}} & 0\\ 0 & 0 & \Delta_{\mathrm{zz}} \end{array}\right) \end{array}$$

As each pedestrian is roughly modelled by a high concentration of 3D points, the final host-to-pedestrian distance estimation error is defined as the mean value of the Δ*_zz_* value corresponding to all 3-D points that lie within the pedestrians detected by the subtractive clustering algorithm.

### 2.5. System parameters

A stereo imaging system needs to know how the various system parameters affect the depth estimation error, especially for automotive applications due to their safety component. Designing a stereo system involves choosing three main parameters: the *focal length* of the cameras, the distance between the cameras (*baseline*) and the *size* of the images. The most important application requirements are the *depth estimation error*, the *runtime* and the distance of the *frontal blind zone*, although the range estimation error is usually the deciding parameter, and the system parameters are chosen in order to meet an acceptable range error. Concerning this topic a considerable number of statistical depth error analysis works have been carried out [7,8] deriving quantitative expressions. Although these expressions describe the relationship between the range error and the system parameters, it is still difficult to obtain a fast method to define those parameters, especially when there are many parameters that affect to the depth estimation error.

**Figure 4.**
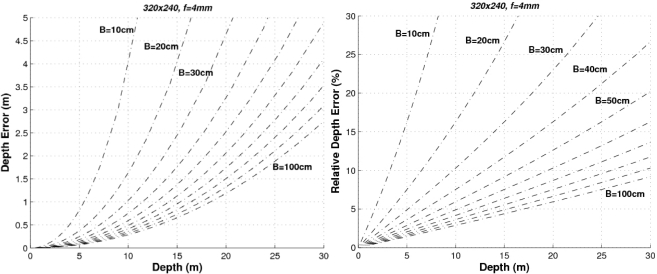
Absolute and relative depth estimation errors for a stereo sensor with *f* = 4 mm and image size of 320 × 240 px, for different baselines.

In order to facilitate the choice of the system parameters we propose the use of pre-computed graphs including different settings. Whereas the H2P distance estimation error is computed assuming the general stereo case (non parallel optical axes), the graphs are computed using the ideal case. From the geometry of a parallel stereo pair (ideal case), *i.e.*, two cameras with parallel optical axes, the same intrinsic parameters (*f_x_*, *f_y_*,*u*_0_, *v*_0_) and separated by a baseline *t_x_*, the depth value of a 3D point *P* = (*X*,*Y*,*Z*)*^T^* can be defined as:

(13) $$Z=f\frac{t_{x}}{x_{r}-x_{l}}=f\frac{t_{x}}{(u_{r}-u_{0})d_{x}-(u_{l}-u_{0})d_{x}}=\frac{f}{d_{x}}\frac{t_{x}}{u_{r}-u_{l}}=f_{x}\frac{t_{x}}{d_{u}}$$

where *f* is the cameras focal length, *x_r_* and *x_l_* are the x-projections in metrics coordinates on the right and left image planes respectively, *d_x_* is the length of a pixel in the x-axis (*f_x_* = *f* / *d_x_*), *u_r_* and *u_l_* are the x-projections in pixel coordinates on the right and left image planes respectively, and *d_u_* represents the disparity value in the x-axis of the image in pixels. Given the baseline *t_x_*, the focal length in pixels in the x-axis *f_x_* and the image size (*W*,*H*), the stereo error can be computed from the maximum disparity *d_u_MAX__*=*W* – 1 (minimum depth) to the minimum disparity *d_u_MIN__* =1 in steps of 1 pixel as follows:

(14) $$\Delta Z=Z_{i}-Z_{i-1}=f_{x}t_{x}\left(\frac{1}{d_{\mathrm{ui}}-1}-\frac{1}{d_{\mathrm{ui}}}\right)=f_{x}t_{x}\frac{1}{d_{\mathrm{ui}}^{2}-d_{\mathrm{ui}}}$$

where the maximum theoretical range is given by *Z_MAX_* = *f_x_t_x_*. Equation (14) describes the relationship between the depth accuracy and the product *f_x_t_x_*, as well as the image size (the higher the image size (*W*,*H*) the higher the disparity range *d_u_MAX__*). In order to provide a graphical representation of the range error we have to fix some parameters. For example, Figure 4 depicts the range error and the relative range error (Δ*Z* / *Z*) for different baselines using images of 320 × 240 pixels and a focal length of 4 mm.

**Figure 5.**
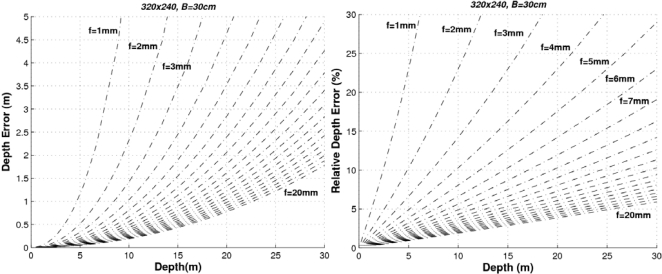
Absolute and relative depth estimation errors for a stereo sensor with *B* = *t_x_* = 400 mm and image size of 320 × 240 px, for different focal lengths.

As can be seen, the higher the baseline the lower the error. Let’s consider that our system (with 320 × 240 images and *f* = 4 mm)) requires a relative error Δ*Z* < 10% up to distances of 20 m. Then the baseline should be greater than 60 cm. If the relative error has to be Δ*Z* < 5% up to distances of 5 m, then the minimum baseline would be 30 cm (and so on). If the baseline is defined to *t_x_* = 300 mm and the images have a size of 320 × 240 pixels, the higher the focal length the lower the error as can be seen in Figure 5.

**Figure 6.**
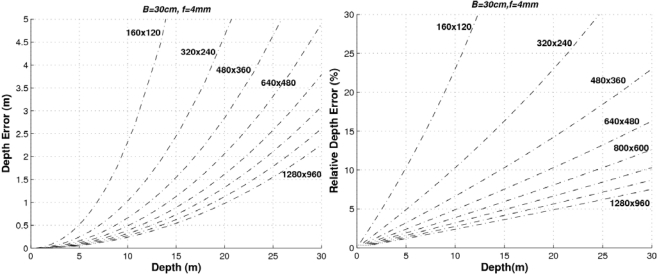
Absolute and relative depth estimation error for a stereo sensor with *B* = *t_x_* = 400 mm and *f* = 4 mm, for different image sizes.

**Figure 7.**
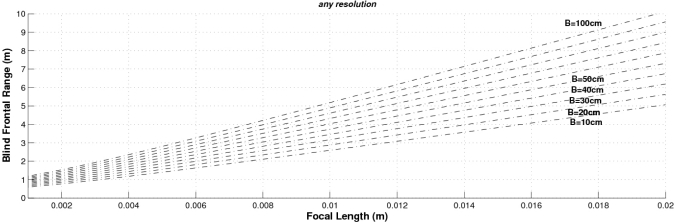
Blind frontal range as a function of the focal length, for different baselines. Note that the size of the images has no effect on the size of the blind frontal area.

**Figure 8.**
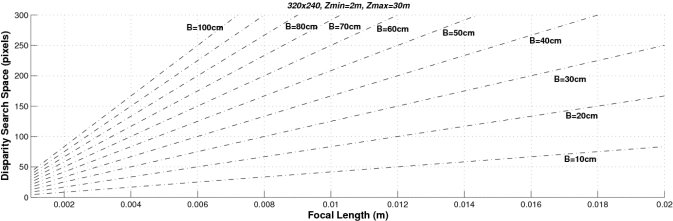
Size of the disparity search space as a function of the focal length, for different baselines with images of 320 × 240 px. The disparity search space is computed from 2 m to 30 m.

**Figure 9.**
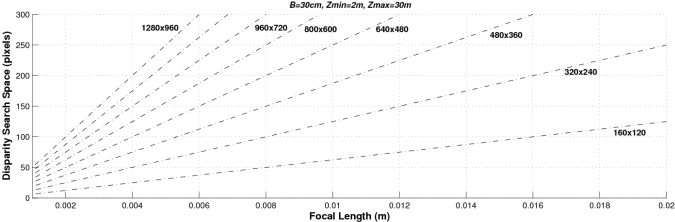
Size of the disparity search space as a function of the focal length, for different image sizes, with a baseline of *B* = *t_x_* = 400 mm. The disparity search space is computed from 2 m to 30 m.

Finally, Figure 6 shows both the absolute and the relative range errors for different image sizes corresponding to a sensor with *f* = 4 mm and *t_x_* = 300 mm. As can be observed, the higher the image size the lower the error. These graphs can be used for determining the system parameters according to the depth error requirements. Accordingly we can conclude that the higher the baseline, the focal length and the size of the images the lower the depth error. However, other parameters have to be taken into account when designing a stereo sensor: the computational load (which is defined by the range of the disparity search space) and the size of the frontal blind zone. As soon as we increase the values of the baseline and the focal length, both the size of the frontal blind zone (see Figure 7) and the range of the disparity search space (see Figure 8) also increase. In addition, the higher the size of the images, the higher the disparity search space (the computational load) as can be seen in Figure 9.

The proposed stereo vision-based pedestrian detection system uses 320 × 240 images, a baseline of *t_x_* = 300 mm and a focal length of *f* = 4 mm. This sensor setup is mainly defined as a trade off between accurate range estimation and low computational load as well as low size of the frontal blind area. We can derive from Figures 4–6 that this sensor setup implies an almost linear relationship (with a slope approximately equal to 1) between the range and the relative range error up to distances of 15 m. As can be observed in Figure 7, the size of the blind frontal area is mainly defined by the focal length of the cameras. In our case, a focal length *f* = 4 mm defines a blind frontal area lower than 1.5 m. Finally, our sensor setup implies a disparity search space of 50 px in a range from 2 m to 30 m, as depicted in Figures 8 and 9, which allows real-time stereo computation. Higher resolutions, e.g., 640 × 480, would certainly produce lower relative depth errors (almost half the error, as can be observed in Figure 6). However, the disparity search space would be increased by a factor of 2 (Figure 9). This is also applicable to the focal length. A focal length of *f* = 8 mm would reduce the relative depth error by a factor 2 up to distances of 15 m (see Figure 5), but the disparity search space would be also increased by a factor of 2 (see Figure 8).

## 3. Experimental Results

**Figure 10.**
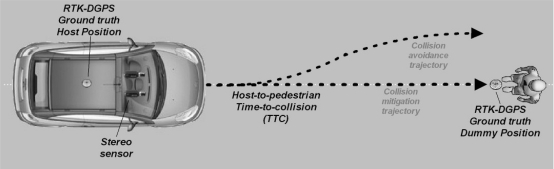
Experimental setup. The RTK-DGPS is used as ground truth data from both pedestrian position and vehicle position. The stereo sensor provides host-to-pedestrian sTTC measurements.

The proposed stereo vision-based pedestrian detection sensor is evaluated in a set of experiments carried out in one of the most challenging tasks in the context of automotive applications: collision avoidance maneuvers. The experimental setup is described in Figure 10. Ground truth data corresponding to both pedestrian position and vehicle position are obtained from the RTK-DGPS (after linear interpolation due to its low sample frequency: 5Hz). First, we locate the DGPS in the dummy position during a few seconds and we obtain its coordinates. Secondly, we install the DGPS sensor onboard the vehicle. Finally, several manually driven avoidance/mitigation maneuvers at different speeds (10, 15, 20, 25 and 30 km/h) are recorded saving data from both the RTK-DGPS and the stereo sensor. Thus, we can compare the results provided by the stereo vision sensor and determine its suitability for this important automotive application.

**Figure 11.**
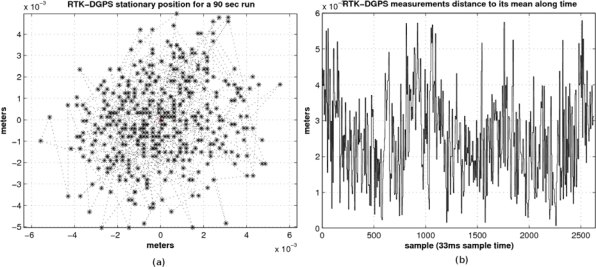
(a) RTK-DGPS stationary position in a 90 s run. (b) RTK-DGPS distance to its mean along time.

In order to support the use of the RTK-DGPS sensor as ground truth we have devised a simple experiment in which we locate the sensor on the dummy position and we obtain the global position during 90 s. Figure 11(a) shows the obtained positions. Figure 11(b) depicts the standard deviation along time with respect to the average value. The maximum deviations in x- and y-axis are 5 mm and 5.6 mm respectively. The standard deviations in x- and y-axis are 0.0036 mm and 0.0041 mm respectively. These values are at least two orders of magnitude lower than the errors obtained from the stereo sensor, supporting the fact that the RTK-DPGS can be used as ground truth data provider (note that the default coverage factor *k* in GPS measurements is 2–3 [15]; commonly *k* = 2 coincides with level of confidence of the interval about 95%. When higher level of confidence is needed, *k* = 3, the level of confidence of the interval is about 99%).

**Figure 12.**
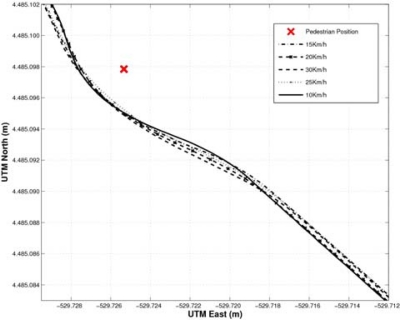
Pedestrian collision avoidance maneuvers at different speeds.

Figure 12, shows the RTK-DGPS trajectories corresponding, where the *x*-axis represents the Universal Transverse Mercator (UTM) East coordinates and the *y*-axis represents the UTM North coordinates in meters.

**Figure 13.**
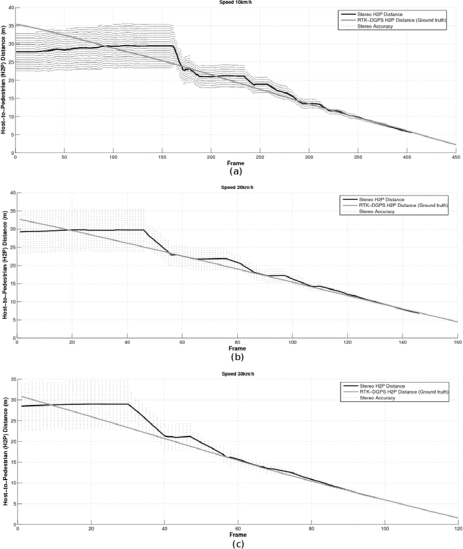
Stereo Host-to-Pedestrian (H2P) distance measurements and their accuracy and RTK-DGPS H2P distance (ground truth) at (a) 10 km/h, (b) 20 km/h and (c) 30 km/h.

In order to compare these trajectories with the ones provided by the stereo sensor, the relative car-to-pedestrian positions with respect to the left camera have to be computed. This transformation is carried out by applying two translations: one from the UTM global reference to the RTK-DGPS onboard the vehicle and other from the DGPS to the left camera. The orientation of both axes is computed using the longitudinal movement of the vehicle. Figures 13a–c depict the host-to-pedestrian distance supplied by the DGPS, with the reference located on the moving vehicle (left camera), and the host-to-pedestrian distance provided by the stereo sensor as well as their quantization errors [Δ*_zz_* from Equation (12)], which are drawn with dotted lines, corresponding to the avoidance maneuver at 10, 20 and 30 km/h respectively.

Some remarkable conclusions can be deduced from these figures. The maximum range (25–30 m) and the inverse proportion between the depth and the stereo accuracy can be easily appreciated (as demonstrated in Section 2.3). The ground truth measurements are almost always (99%) inside the limits of the stereo measurements plus their corresponding quantization errors, which proves that the stereo sensor provides information accurate enough despite its inner accuracy constraints. The reason why there are some cases where the H2P ground truth measurements are outside the error interval is because the stereo quantization errors are not computed according to the filtered values (note that the Kalman filter blocks high frequency changes) but to the measurements [e.g., see frame number 30 in Figure 13(c)]. Although stereo depth measurements are not reliable at long distances, their accuracy improves in proportion to the collision risk, *i.e.*, as the car-to-pedestrian distance decreases. For example, at 15 m the depth error is about ±1.5 m, at 10 m is about ±0.7 m and at 5 m is lower than ±0.2 m. As suggested in [16] for braking maneuvers, we deduce that for avoidance maneuvers the decision to start the avoidance maneuver may well be based on TTC information as directly available to the driver from the optic flow field. This TTC information is an important cue for the driver in detecting potentially dangerous situations.

**Figure 14.**
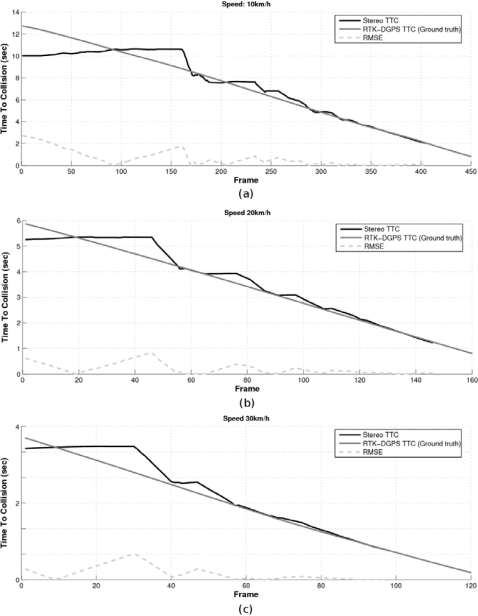
RTK-DGPS TTC, stereo TTC and absolute error in avoidance experiments performed at (a) 10 km/h, (b) 20 km/h and (c) 30 km/h.

Figures 14a–c shows the TTC computed by means of the RTK-DGPS and the stereo sensor as well as the corresponding absolute error in the experiment performed at 10, 20 and 30 km/h respectively. The error is clearly unacceptable for TTC values above 8 s. However, the accuracy of the measurements increases as long as the TTC decreases.

In Table 1 we show the root mean square error (RMSE) of the TTC for all the speeds, specifying the error for TTC lower than 8 and 4 s. On average, the error for TTC < 8 s is lower than 0.3 s and for TTC < 4 s is lower than 0.1 s. In addition, we can see that the larger the speed the larger the error, although this relationship is not linear. A possible explanation for this may be the influence of other camera settings such as the exposure time. In addition, the Kalman filter performance can be reduced since the higher the speed the lower the amount of measurements available.

## 4. Conclusions

This paper presents an analytical study of the depth estimation error of a stereo vision-based pedestrian detection sensor for automotive applications such as pedestrian collision avoidance and/or mitigation. The sensor comprises two synchronized and calibrated low-cost cameras, providing information about the relative pedestrian position with respect to the host vehicle (H2P distance) and the TTC. Pedestrians are detected in a six stage process: *non-dense reconstruction, pitch estimation, 3D clustering, 2D classification, tracking and decision making.*

The accuracy of the measurements provided by the proposed sensor is obtained by computing the stereo quantization error. Sensor setup is defined according to the application requirements. The relationship between the relative range error and the sensor parameters (focal length, baseline and images size) is analyzed by means of graphs.

The proposed sensor is validated in a set of experiments in which real collision avoidance maneuvers were carried out by real drivers and with real pedestrians up to speeds of 30 km/h. The experimental results demonstrate that the sensor provides suitable measurements despite its inner accuracy constraints due to the quantization error. Even the fact that sensors measurements (H2P distance and TTC) are not reliable at long distances, their quantization errors decrease as long as both the distance and the TTC decrease. In other words, the higher the risks, the better the sensor accuracy.

These statements can be accepted up to speeds of 30 km/h. The risks associated with performing collision avoidance maneuvers at higher speeds are not acceptable with the current experimental setup. However, one main conclusion can be extrapolated from our results: higher speeds will endure higher errors in the estimated TTC, compromising the effectiveness of the proposed approach. In order to increase the accuracy of the measurements provided by the stereo sensor, higher resolution images and longer baseline can be used. However, that would increase the computational cost.

The performed field test provided encouraging results and proved the validity of the proposed sensor for being used in the automotive sector towards applications such as autonomous pedestrian collision avoidance.

## Figures and Tables

**Table 1.** RMSE of the TTC.

**Speed**	**RMSE (s)**	**RMSE (s) (TTC < 8 s)**	**RMSE (s) (TTC < 4 s)**

10 km/h	0.9625	0.2164	0.0341
15 km/h	0.8775	0.3419	0.0360
20 km/h	0.5430	0.2200	0.0919
25 km/h	0.5997	0.2870	0.1281
30 km/h	0.7110	0.3731	0.1436

**Average**	0.7387	0.2877	0.0867
